# Genetic and evolutionary characterization of RABVs from China using the phosphoprotein gene

**DOI:** 10.1186/1743-422X-10-14

**Published:** 2013-01-07

**Authors:** Lihua Wang, Hui Wu, Xiaoyan Tao, Hao Li, Simon Rayner, Guodong Liang, Qing Tang

**Affiliations:** 1 State Key Laboratory for Infectious Disease Prevention and Control, Institute for Viral Disease Control and Prevention, Chinese Center for Disease Control and Prevention, 155 Changbai St., Changping Dist, Beijing, 102206, China; 2 State Key Laboratory for Virology, Wuhan Institute of Virology, Chinese Academy of Sciences, Hubei, 430071, China

**Keywords:** Rabies virus, Phosphoprotein gene, Genetic diversity, Molecular evolution

## Abstract

**Background:**

While the function of the phosphoprotein (P) gene of the rabies virus (RABV) has been well studied in laboratory adapted RABVs, the genetic diversity and evolution characteristics of the P gene of street RABVs remain unclear. The objective of the present study was to investigate the mutation and evolution of P genes in Chinese street RABVs.

**Results:**

The P gene of 77 RABVs from brain samples of dogs and wild animals collected in eight Chinese provinces through 2003 to 2008 were sequenced. The open reading frame (ORF) of the P genes was 894 nucleotides (nt) in length, with 85-99% (80-89%) amino acid (nucleotide) identity compared with the laboratory RABVs and vaccine strains. Phylogenetic analysis based on the P gene revealed that Chinese RABVs strains could be divided into two distinct clades, and several RABV variants were found to co circulating in the same province. Two conserved (CD1, 2) and two variable (VD1, 2) domains were identified by comparing the deduced primary sequences of the encoded P proteins. Two sequence motifs, one believed to confer binding to the cytoplasmic dynein light chain LC8 and a lysine-rich sequence were conserved throughout the Chinese RABVs. In contrast, the isolates exhibited lower conservation of one phosphate acceptor and one internal translation initiation site identified in the P protein of the rabies challenge virus standard (CVS) strain. Bayesian coalescent analysis showed that the P gene in Chinese RABVs have a substitution rate (3.305x10^-4^ substitutions per site per year) and evolution history (592 years ago) similar to values for the glycoprotein (G) and nucleoprotein (N) reported previously.

**Conclusion:**

Several substitutions were found in the P gene of Chinese RABVs strains compared to the laboratory adapted and vaccine strains, whether these variations could affect the biological characteristics of Chinese RABVs need to be further investigated. The substitution rate and evolution history of P gene is similar to G and N gene, combine the topology of phylogenetic tree based on the P gene is similar to the G and N gene trees, indicate that the P, G and N genes are equally valid for examining the phylogenetics of RABVs.

## Introduction

Rabies is a lethal neurological disease caused by infection with members of the genus *lyssavirus*. Eleven distinct lyssavirus species are currently recognized worldwide [1]. In China, only the classical rabies virus (RABV) is known to circulate in dogs, which serve as the principal reservoir and transmitter of rabies to humans and domestic animals [2,3]. RABV has a non-segmented negative sense RNA genome comprised of five genes in the order 3’-N-P-M-G-L-5’ [4]. The relatively divergent P gene [5-7] encodes a multifunctional phosphoprotein (P protein) [8] and has been extensively investigated using laboratory adapted RABV strains. Five serine residues of the challenge virus standard (CVS) strain have been identified as phosphate acceptor sites [9]. Also, P is a critical component of the viral polymerase responsible for transcription and replication through its binding to the N and L proteins [10-12]. Two independent N binding sites, one located within amino acids (aa) 66–176 at the N-terminal half of the protein and the other located to amino acids 268–297 within 50 residues of the C-terminus, have been found in the P protein [10,11]. Via N-P complexes, the nonspecific aggregation of N can be prevented and can keep N in a suitable form for specific encapsidation [13]. The short lysine-rich motif FSKKYKF (aa 214–220) is an important component of the C-terminal N protein binding domain of P [14]. P is associated with the genome expression process by acting as an intermediary for the attachment of the L polymerase core to the N-RNA template [15]. In addition, the first 19 N-terminal residues of P confer L protein binding [10]. P also specifically interacts with many host cell components. It has been reported that the sequence (K/R)XTQT represents a conserved cytoplasmic dynein light chain (LC8) binding motif, an element of the microtubule-associated motors involved in minus-end directed axonal transport, through which it may play some role in viral retrograde transport [16-18]. P interferes with the host’s innate immune system through inhibition of the activities of interferon regulatory factor 3 (IRF3) [19] and signal transducer and activator of transcription 1 (STAT1) [20,21], thereby abrogating the cellular type 1 interferon pathway. P also binds to the promyelocytic leukemia (PML) protein, which has many possible functions in nuclear trafficking, viral defense mechanisms and apoptosis [22], suggesting that P acts an antagonist towards antiviral PML function [23].

Since all functional studies on the RABV P protein have been performed using a limited number of laboratory strains, the relevance of the results to field isolates is unclear. In this study we sequenced the P gene of Chinese RABV street strains collected in most rabies endemic areas of China and investigated the genetic diversity, sequence characteristics and estimated the overall substitution rate of the P gene. In addition, the phylogeny and evolution history of Chinese RABVs based on P gene were examined.

## Results

### Length and identity of P gene in Chinese RABV street strains

**Figure 1:**
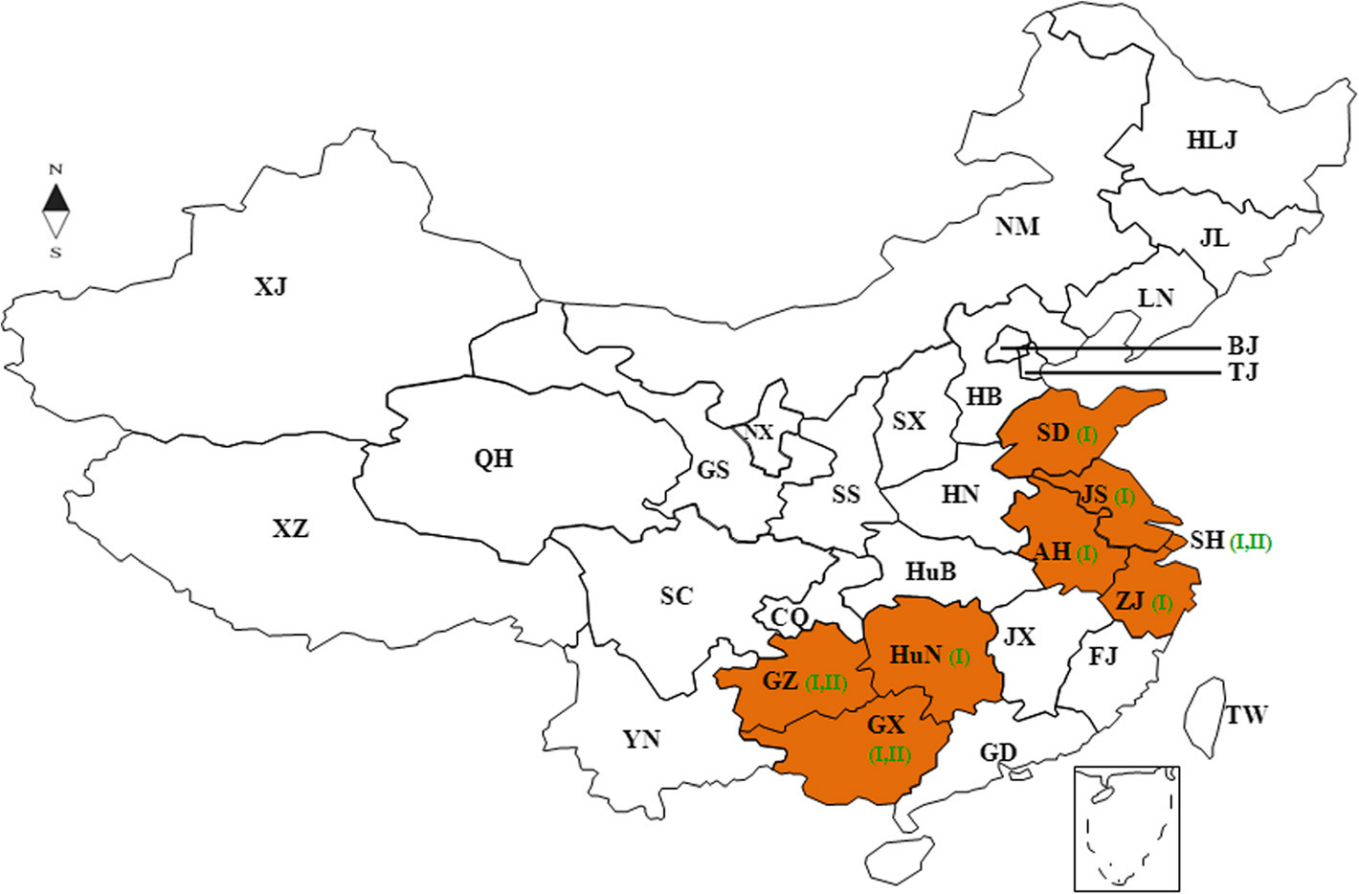
**Locations of specimen collection in this study.** AH, GX, GZ, HuN, JS, SD, SH, ZJ, indicate Anhui, Guangxi, Guizhou, Hunan, Jiangsu, Shandong, Shanghai, Zhejiang provinces of China, where the specimen were collected in this study during 2003 to 2008. I and II indicate the presence of isolates corresponding to Clade I and II as classified by the phylogenetic tree.

77 RABV positive brain specimens were detected by direct fluorescent antibody (DFA) and subjected to RT-PCR for determination of the P gene of RABV street strains. These specimens were from field captured dogs and ferret badgers in eight provinces which had high (Guangxi, Guizhou and Hunan provinces), middle (Jiangsu and Shandong provinces) and low (Anhui, Shanghai, and Zhejiang provinces) incidences of rabies (Figure 1). The open reading frame (ORF) of the P gene, corresponding to nt 1514–2407 of the PV strain (M13215), was determined for all 77 RABV isolates. The ORF of the P gene of all Chinese RABVs were 894 nt in length and sequences were submitted to GenBank (HM582519–HM582595). The species of origin, the year of isolation, and geographical location of these sequences are summarized in Table 1.

**Table 1:** Background information of P gene sequences used in this study

**Genus/isolates**	**Host**	**Origin**	**Year**	**GenBank acc. no.**	**Genus/isolates**	**Host**	**Origin**	**Year**	**GenBank acc. no.**
***AH8***	Dog	AH	2005	HM582562	***SH9***	Dog	SH	2004	HM582564
***AH12***	Dog	AH	2005	HM582567	***SH15***	Dog	SH	2004	HM582555
***GX0801***	Dog	GX	2008	HM582588	***SH16***	Dog	SH	2004	HM582584
***GX0802***	Dog	GX	2008	HM582589	***SH17***	Dog	SH	2004	HM582554
***GX0803***	Dog	GX	2008	HM582590	***SH19***	Dog	SH	2004	HM582550
***GX0804***	Dog	GX	2008	HM582591	***SH20***	Dog	SH	2004	HM582532
***GX0805***	Dog	GX	2008	HM582592	***SH24***	Dog	SH	2003	HM582536
***GX0806***	Dog	GX	2008	HM582593	***SH25***	Dog	SH	2003	HM582553
***GX0807***	Dog	GX	2008	HM582594	***SH27***	Dog	SH	2003	HM582529
***GX0809***	Dog	GX	2008	HM582595	***SH28***	Dog	SH	2003	HM582524
***GX1***	Dog	GX	2006	HM582521	***SH29***	Dog	SH	2003	HM582548
***GX2***	Dog	GX	2006	HM582546	***SH30***	Dog	SH	2003	HM582549
***GX3***	Dog	GX	2006	HM582525	***SH31***	Dog	SH	2003	HM582556
***GX5***	Dog	GX	2006	HM582571	***SH32***	Dog	SH	2003	HM582561
***GX6***	Dog	GX	2006	HM582581	***D03***	Dog	ZJ	2008	HM582568
***GX7***	Dog	GX	2006	HM582526	***D05***	Dog	ZJ	2008	HM582569
***GX8***	Dog	GX	2005	HM582522	***D06***	Dog	ZJ	2008	HM582573
***GX9***	Dog	GX	2006	HM582535	***D07***	Dog	ZJ	2008	HM582570
***GX10***	Dog	GX	2005	HM582523	***D10***	Dog	ZJ	2008	HM582586
***GX16***	Dog	GX	2005	HM582538	***F03***	CFB	ZJ	2008	HM582587
***GX18***	Dog	GX	2006	HM582527	CGX0521	Dog	GX	2005	EU004759
***GX19***	Dog	GX	2006	HM582543	CGX0603	Dog	GX	2006	EU004755
***GX24***	Dog	GX	2005	HM582547	CGX0614	Dog	GX	2006	EU004758
***GX26***	Dog	GX	2006	HM582528	CHdg18	Dog	GX	2007	AB458796
***GZ1***	Dog	GZ	2005	HM582580	GX4	Dog	GX	1994	GU358653
***GZ3***	Dog	GZ	2005	HM582544	HN10	Human	HN	2006	EU643590
***GZ6***	Dog	GZ	2005	HM582579	CHN0635	Human	HN	2006	EU004777
***GZ8***	Dog	GZ	2005	HM582531	CJS0523	Dog	JS	2005	EU004782
***GZ9***	Dog	GZ	2005	HM582540	JX08-45	CFB	JX	2008	GU647092
***GZ10***	Dog	GZ	2005	HM582541	NeiMeng925	Dog	NM	2008	FJ415313
***GZ11***	Dog	GZ	2005	HM582562	SH06	Dog	SH	2006	GU345748
***GZ12***	Dog	GZ	2005	HM582545	SH26	Dog	SH	2003	HM582583
***GZ13***	Dog	GZ	2005	HM582537	D01	Dog	ZJ	2008	FJ712193
***GZ14***	Dog	GZ	2005	HM582551	D02	Dog	ZJ	2008	FJ712194
***GZ15***	Dog	GZ	2005	HM582552	D04	Dog	ZJ	2008	FJ032321
***GZ16***	Dog	GZ	2005	HM582534	D08	Dog	ZJ	2008	FJ032322
***GZ17***	Dog	GZ	2005	HM582530	F02	CFB	ZJ	2008	FJ712195
***GZ21***	Dog	GZ	2008	HM582572	8743THA	Human	Thailand	1983	EU293121
***HN4***	Dog	HuN	2005	HM582519	8764THA	Human	Thailand	1983	EU293111
***HN27***	Dog	HuN	2005	HM582582	INRV	Human	India	2005	AY956319
***HN29***	Dog	HuN	2005	HM582520	NNV-RAB-H	Human	India	2006	EF437215
***HN30***	Dog	HuN	2005	HM582542	CVS	Challenge virus standard			X55727
***JS29***	Dog	JS	2006	HM582563	aG	Vaccine st rain	China		DQ646875
***JS34***	Dog	JS	2006	HM582565	CTN	Vaccine st rain	China		FJ959397
***SD1***	Dog	SD	2008	HM582557	PV	Vaccine st rain	France		M13215
***SD7***	Dog	SD	2007	HM582559	SADB19	Vaccine st rain	USA		M31046
***SD8***	Dog	SD	2007	HM582558	Ni-CE	Vaccine st rain	Japan		AB128149
***SD10***	Dog	SD	2007	HM582533	RC-HL	Vaccine st rain	Japan		AB009663
***SD11***	Dog	SD	2007	HM582575	Flury-HEP	Vaccine st rain	USA		GU565704
***SD12***	Dog	SD	2007	HM582574	8619NGA	Bat	Nigeria	1956	EU293110
***SD13***	Dog	SD	2007	HM582576	MOKV	Cat	Zimbabwe	1981	NC006429
***SD14***	Dog	SD	2006	HM582577	86132SA	Human	South Africa	1971	EU293119
***SD23***	Dog	SD	2008	HM582578	8918FRA	Bat	France	1989	EU293112
***SH1***	Dog	SH	2005	HM582585	9018HOL	Bat	Netherlands	1986	EU293114
***SH5***	Dog	SH	2005	HM582566	ABLV	Bat	Aust ralia	1996	NC003243
***SH7***	Dog	SH	2004	HM582560	***WCBV***	Bat	Russia	2002	EF614258

**Note:** New sequences in this study are labeled **Bold and italic;** AH, GX, GZ, HuN, JS, JX, NM, SD, SH, ZJ, indicate Anhui, Guangxi, Guizhou, Hunan, Jiangsu, Jiangxi, Inner Mongolia, Shandong, Shanghai, Zhejiang provinces of China, respectively; CFB: Chinese Ferret Badgers.

The P gene of all the Chinese RABVs encodes a 297 amino acid protein identical in length to the P gene in the PV vaccine strain (M13215). The nucleotide and deduced amino acid sequences were aligned and compared with the sequences of laboratory, street and vaccine strains. Among the 77 Chinese RABVs isolates, the nucleotide and amino acid sequence identities of the P gene were 80.2-100% and 85.2-100% respectively. When compared with the vaccine strains, the P gene of the 77 Chinese RABVs had 85.0-99.2% (80.0-89.5%) amino acid (nucleotide) identity, respectively.

### Variation of functionally significant sequence motifs and residues

**Figure 2:**
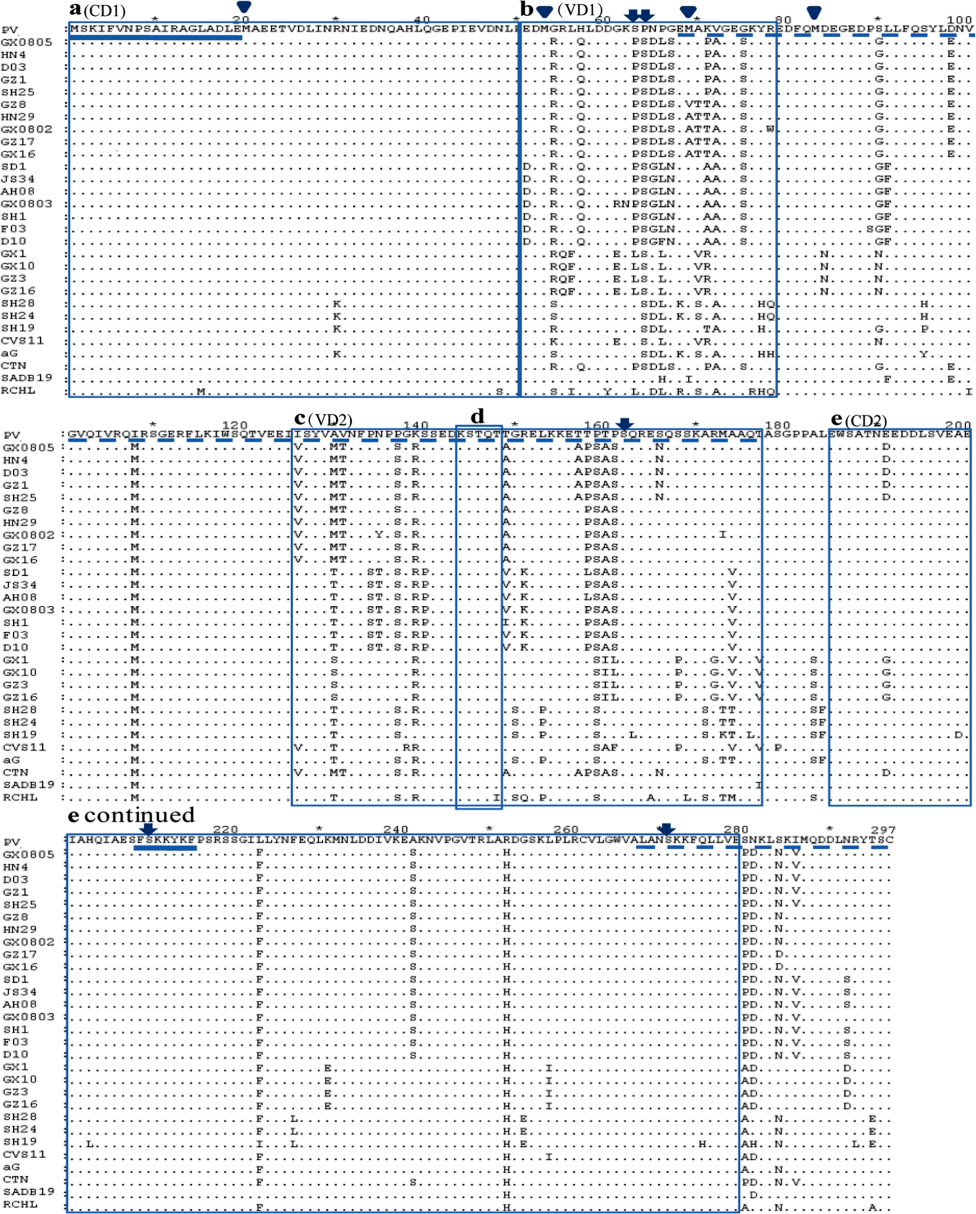
**Alignment of the P amino acid sequences of street strains collected in this study, vaccine strains and standard challenge virus strain CVS11.** Dots indicate amino acids that are in agreement with the reference sequence (PV vaccine strain (M13215)) on the first line. Box **a** and **e**: conserved domains 1 and 2; box **b** and **c**: variable domains 1 and 2; box **d**: Dynein light chain (LC8)-binding motif; solid underline shows L protein binding region(1–19 aa) and the lysine-rich motif (209–216 aa), respectively; dashed underline shows N protein binding site; triangles indicate the positions of methionine residues and confirmed translation initiation in the CVS strain; arrows indicate the positions of serine residues identified as phosphoacceptors in the P protein of the CVS strain.

Based on the identity analysis, an amino acid alignment of the 77 Chinese RABVs isolates and representative sequences of laboratory and vaccine strains was generated and investigated for mutations (Figure 2). In total, seventy two amino acid substitutions throughout the P protein were observed in the Chinese RABVs isolates relative to the PV vaccine strain (M13215). Based on the location of the mutations, the protein had both highly conserved and highly variable regions that have been previously shown to be associated with viral function. Specifically, there were two conserved domains at residues 1–50 (CD1) and 184–279 (CD2) and two variable domains at residues 51–80 (VD1) and 126–178 (VD2) (Figure 2). The first 19 aa residues at the N-terminal, shown to be associated with L binding [10], are completely conserved. The short lysine-rich segment FSKKYKF (209-216aa) thought to be an important component of the C-terminal N protein binding domain [14], is also highly conserved in all Chinese isolates. Within region VD2, the cytoplasmic dynein LC8 binding motif (K/R) XTQT [18] is conserved with Chinese RABVs, and all the strains contain the motif KSTQT (located between 144 and 148 aa). Interestingly, the STAT-1 binding sites, located in the last 30 aa residues of the C-terminal [20] showed limited conservation in Chinese isolates. The internal translation initiation sites 20, 53, 69, and 83 in the P protein of the rabies challenge virus standard (CVS) strain [24] are at the same position in the Chinese RABVs isolates. Three of them (Met_20_, Met_53_, and Met_83_) are completely conserved in Chinese RABVs. For the remainder, the mutation Met_69_ toVal_69_ occurred in isolate GZ8 and mutation Met_69_ to Ala_69_ occurred in isolates HN29, GX0802, GZ7, GX16. Four (Ser_64_, Ser_162_, Ser_210_, Ser_271_) of five serine residues reported to function as phosphate acceptors in the P protein of the rabies challenge virus standard (CVS) strain [9] were absolutely conserved. For the fifth residues mutation Ser_63_ to Phe_63_ or Ser_63_ to Leu_63_ was observed in all the Chinese isolates with the exception of isolate SH19.

### Phylogenetic analysis of RABVs in China

**Figure 3:**
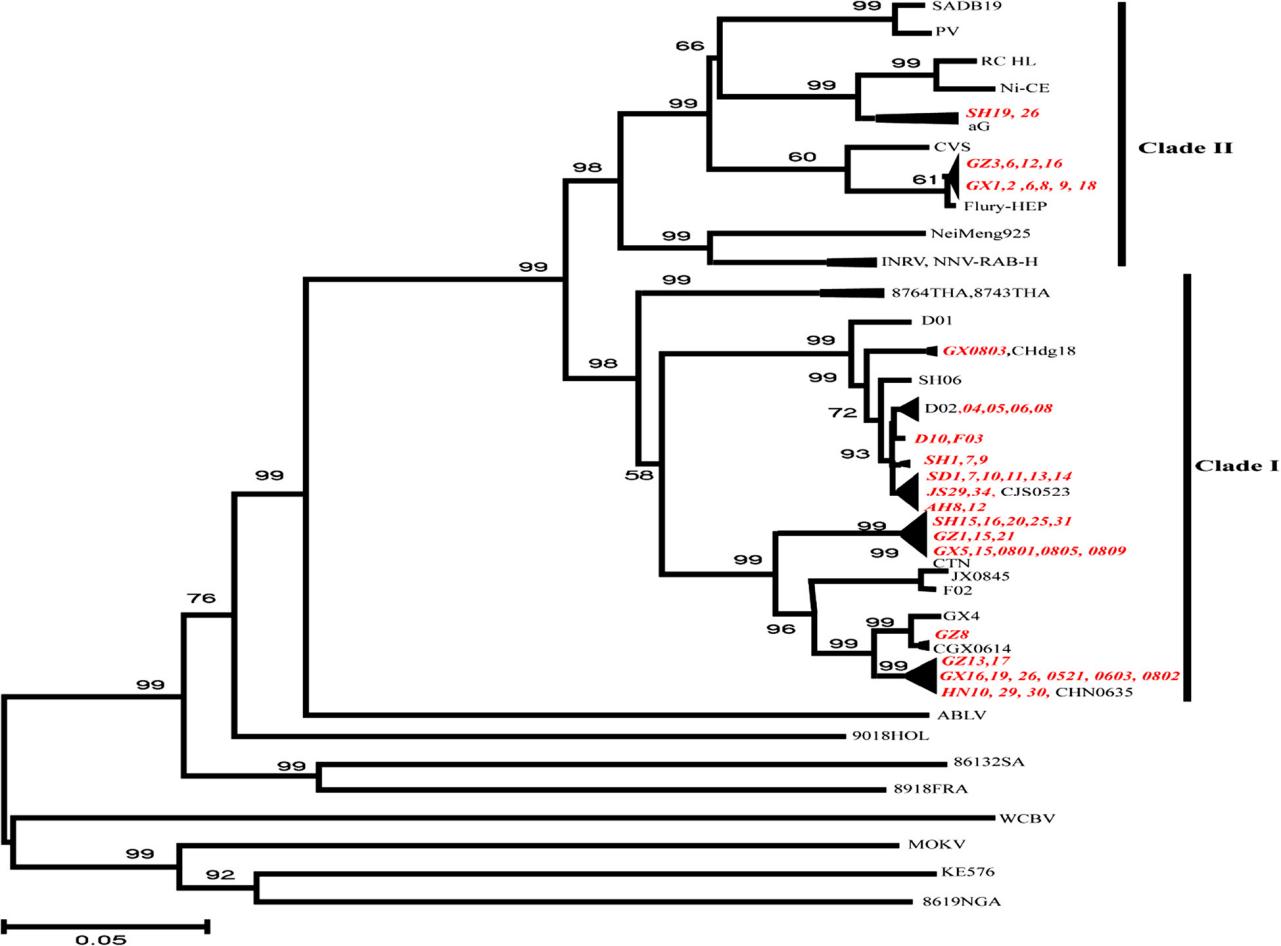
**Neighbor-joining phylogenetic tree (P-distance) for the P gene of RABVs collected in this study, vaccine strains and representative strains of *lyssavirus*.** Numbers indicate the bootstrap value from 1000 replicates. Clade I and clade II are indicated.

A phylogenetic analysis of 113 (77 collected in this study, with an additional 36 samples downloaded from GenBank) RABV P gene sequences was performed. The Neighbor-joining tree is shown in Figure 3 with bootstrap values shown for the main groupings. The sequences of Chinese isolates were divided into two major clades, named clade I and II (Figure 3). Most of the 77 isolates collected in this study are placed in Clade I (bootstrap value = 98). These isolates are mainly from Anhui, Guangxi, Guizhou, Hunan, Jiangsu, Shandong, Shanghai and Zhejiang provinces, and show a close evolutionary history with the RABVs isolates from Thailand (8764THA, EU293111; 8743THA, EU293121). Clade II (bootstrap value = 98) are composed of isolates from Shanghai, Guizhou and Guangxi provinces, are grouped with the standard challenge strain (CVS) and vaccine strains (aG, PV, RC HL, SADB19, Ni-CE and Flury-HEP), and show a close relationship to arctic-related RABVs strains from India and northeastern China (Inner Mongolia). Chinese Ferret badger strain F03 was grouped with D10 strain isolated from dog in the same location, indicating that RABVs spillover can occur between dogs and Chinese Ferret badgers.

### Substitution rates and evolution history analysis of P gene

**Figure 4:**
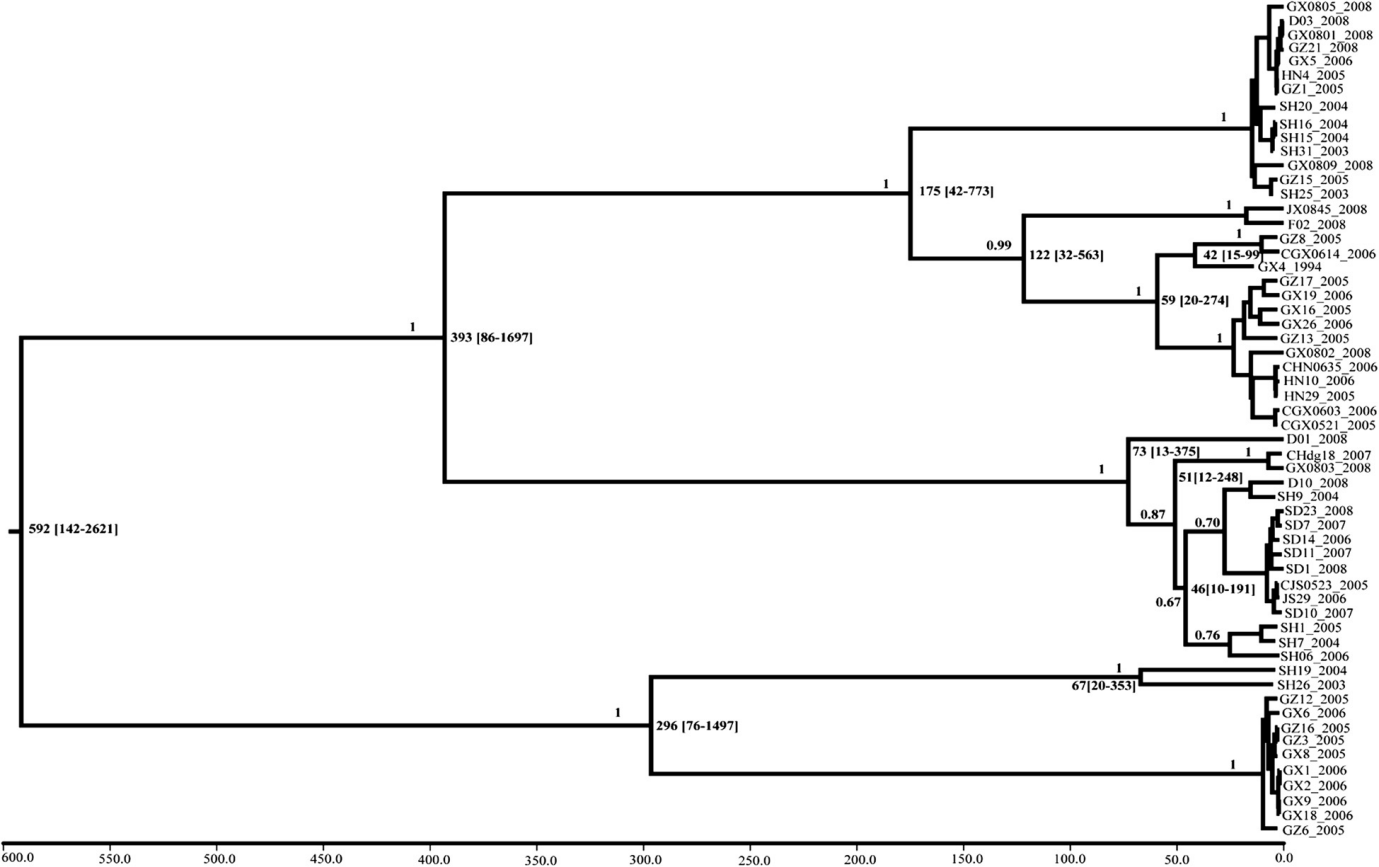
**Maximum clade credibility (MCC) tree from Bayesian coalescent analysis based on a subset of the P gene sequences in this study.** The estimated TMRCA for this dataset and its 95% HPD values are indicated. Isolate names are given according to Table 1. Horizontal branches are drawn to a scale of estimated year of divergence, with tip times reflecting sampling date (year). Posterior probability values for all of the major nodes are shown. See materials and methods for details.

By using a Bayesian Markov chain Monte Carlo method, the evolutionary history, including evolutionary rates of populations (nucleotide substitutions per site per year) and TMRCA (the most recent common ancestor) were analyzed based on 58 P gene sequences (Only sequences with an homology less than 98% and with full background information in terms of location and isolation time were used in the calculation). The estimated mean rate of nucleotide substitution for the P gene of Chinese RABVs was 3.305x10^-4^ substitutions per site per year (95% HPD values, 1.127-6.209x10^-4^ substitutions per site per year). Bayesian coalescent analysis estimated the most recent common ancestor (TMRCA) to have originated 592 years ago (95% HPD, 142–2621 years) (Figure 4).

## Discussion and conclusion

The N gene (the most conserved and abundant mRNA in infected cells) and G gene (plays a crucial role in viral neurotropism and pathogenicity) have been widely targeted for genetic, molecular epidemiology and evolutionary analysis of RABVs [4,25-28]. In contrast, for the P gene, only a few laboratory [29,30] and wild-type RABV strains [31], an ABLV isolate [32] and Mokola virus [33] have been genetically characterized. In this study we attempted to characterize the genetic and evolutionary properties of the P gene of Chinese street RABVs. 77 P genes from brain samples of dogs and wild animals in eight provinces through 2003 to 2008 were sequenced and subjected to molecular and phylogenetic analysis.

Several substitutions were found in the Chinese RABVs strains compared to the laboratory adapted and vaccine strains. The nucleotide (≥ 80.2%) and amino acid sequence identities (≥ 85.2) of the P gene were lower than the corresponding values for the N (≥87.6% and 95.4%) and G gene (≥87% and 93.8%) [26,28]. Consistent with the wild type RABVs strains isolated in North America [31], two conserved (CD1, 2) and two variable (VD1, 2) domains were identified in Chinese RABVs. The observed substitutions are mainly located in the middle of P, while the N and C terminal are relatively well conserved. As reported previously, the need to retain overall negative charge rather than primary sequence would explain the VD1 region’s high level of diversity [6]. The poorly conserved VD2 might indicate a function as a spacer/hinge segment analogous to the hinge region of the P gene in Vesicular stomatitis virus (VSV) located between two functionally important domains [34]. Two sequence motifs, one believed to confer binding to the cytoplasmic dynein light chain LC8, and a lysine-rich sequence probably contributing to N protein binding [14], were conserved throughout Chinese RABVs samples, while the STAT-1 binding sites [20], internal translation initiation sites and phosphate acceptor sites showed different degrees of variation. Whether these variations could affect the biological characteristics of Chinese RABVs need to be further investigated.

There have been several previous estimates of RABVs substitution rates for the G gene (1.2-6.5 x10^-4^ substitutions per site per year) and the N gene (1.1-5.6 x10^-4^ substitutions per site per year) based on dog, fox and mongoose RABVs samples collected worldwide [25,27,35-38]. In this study, Bayesian coalescent analysis showed that mean substitution rate of the P gene for the Chinese RABVs isolates is 3.305×10^-4^ substitutions per site per year, which indicates that the genome RNA of RABVs circulating worldwide is stable. The TMRCA of cosmopolitan canine RABV variants has previously been estimated to be between 284 and 504 years ago [39]. The mean divergence time estimated based on the the G gene is 583 years ago for RABVs circulating globally [25,35], and 596 years ago for RABVs for current Chinese RABVs [27]. Using a similar analysis, we estimated the average TMRCA of RABVs circulating in China based on the P gene to be 592 years ago, which was in accordance with previous reports for RABVs.

Previous phylogenetic studies based on the G and N genes [26,28,39,40] showed that RABVs in China can be classified into distinct clades or groups. The phylogenetic analysis in this report based on the P gene revealed that Chinese RABVs could be divided into two distinct clades, and that isolates from more than one clade RABV variants are currently co-circulating in the same Chinese provinces. Also, RABVs in Clades I are grouped with RABVs from Thailand, and RABVs in clade II are grouped with RABVs from India. The topology of the phylogenetic tree based on the P gene is similar to the G and N gene trees [26,28,39,40]. This indicates that the P, G and N genes are equally valid for examining the phylogenetics of RABVs and is consistent with observations that the N, P, M, G and L genes of RABVs interact and evolve in a cooperative manner to effect virus infection and evolution [41,42].

## Methods

### Viral specimens sampling

Brain specimens were collected as part of a national surveillance program from dogs used as meat in restaurants and from suspected rabid Ferret badgers from eight provinces (Anhui, Guangxi, Guizhou, Hunan, Jiangsu, Shandong, Shanghai and Zhejiang) in China from 2003 to 2008 (Figure 1).

### Detection and sequencing of RABV

All specimens were examined by using a direct immune fluorescence assay (DFA) [26] with a fluorescent-labeled monoclonal antibody against the RABV N protein (Rabies DFA Reagent; Chemicon Europe Ltd., Chandlers Ford, UK). For all identified RABV specimens, RNA was extracted from tissue of rabies-infected brains (0.1 g) with TRIzol Reagent (Invitrogen, Carlsbad, CA, USA) and used as template for cDNA synthesis with Ready-To-Go You-Prime First-Strand Beads (Amersham Pharmacia Bioscience, Chalfont St. Giles, UK) and a rabies P gene specific primer: Pfor 5’-GAACCATCCCAAAYATG AG -3’ (corresponding to bases 1500–1519 of the positive sense genome sequence of the PV strain). The ORF sequence of the P gene, encoding regions corresponding to bases 1514 to 2407 of the total genetic sequence of the PV strain, was amplified with primers Pfor and Prev 5’- CTATCTTGCGCAGAAARTTCAT -3’ (corresponding to bases 2496 to 2517 of the positive sense genome sequence of the PV strain). PCR products were purified by using the QIAquick PCR Purification Kit (QIAGEN Ltd., Crawley,UK) and sequenced with an ABI PRISM 3100 DNA sequencer (Applied Biosystems, Foster City, CA, USA).

### Sequence alignment and phylogenetic analysis

P gene sequences of lyssaviruses deposited in GenBank were downloaded and combined with the newly sequenced samples to form the dataset used in this study. Alignment of nucleotide sequences and deduced amino acid sequence were performed by using the ClustalX program, version 2.1 [43]. Genetic identities were determined using the Bio-Edit program [44] and MegAlign software version 5 (DNAStar, Inc., Madison,WI, USA). Phylogenetic and evolutionary analyses were conducted using Mega 3.1 [45]. Neighbor-joining (NJ) phylogenetic trees were constructed using evolutionary distance correction statistics [46,47]. Bootstrap analysis was performed using 1000 replications and values greater than 70% were regarded as strong evidence for particular phylogenetic groupings.

### Bayesian Markov chain Monte Carlo (MCMC) evolutionary analysis

Evolutionary history, including evolutionary rates of populations (nucleotide substitutions per site per year) and TMRCA (the most recent common ancestor) were inferred by using the Bayesian Markov chain Monte Carlo (MCMC) method available in the BEAST software package (http://beast.bio.ed.ac.uk/Main_Page)[48]. Briefly, an input file for BEAST was generated by using the BEAUti program with sequences dated according to the year of isolation. Sequences with homology greater than 98% were removed from the analysis using TCOFFEE. The best-fit model of nucleotide substitution for Bayesian analysis was selected with Modeltest 3.7 [49]. The general time reversible (GTR) substitution model, incorporating a proportion of invariable sites (I) and a gamma distribution of rate variation among sites (C4) was used for the BEAST analysis. Both strict and relaxed (uncorrelated exponential and lognormal) molecular clocks [50] were considered to explore the extent of variation in the rate of nucleotide substitution. The BEAST output was assessed using the TRACER program. The maximum clade credibility (MCC) tree was generated using Figtree (available from http://beast.bio.ed.ac.uk).

## Competing interests

The authors declare that they have no competing interests.

## Authors' information

Dr. Lihua Wang, Ph.D., is an associate professor at the State Key Laboratory for Infectious Disease Prevention and Control, the Institute for Viral Disease Control and Prevention, Chinese Center for Disease Control and Prevention. His current research focuses on molecular epidemiology of Rabies virus, development reverse genetic system of rabies virus and basic research related to rabies.

## Authors’ contributions

LHW did genetic mutation, phylogenetic and evolution analysis and drafted the manuscript; HW carried out nucleic acid detection and sequencing; XYT and HL participated in the collection of samples; SR participated the genetic mutation, phylogenetic and evolution analysis; GDL participated in the design of experiments; QT conceived of the study, and participated in its design and coordination. All authors read and approved the final manuscript.

## References

[B1] International Committee on Taxonomy of Viruses ICTV files and discussions. ICTV master species list 2009 Version 4. http://talk.ictvonline.org/files/ictvdocuments/m/msl/1231.aspx (accessed 02.06.10)

[B2] Tang XC Luo M Zhang SY Anthony RF Hu RL Pivotal role of dogs in rabies transmission, China Emerg Infect Dis 2005 11 12 1970 1972 10.3201/eid1112.050271 16485494 PMC3367627

[B3] Hu RL Tang Q Tang JR Fooks AR Rabies in China: an update Vector Borne Zoonotic Dis 2009 9 1 1 11 10.1089/vbz.2008.0046 18803503

[B4] Delmas O Holmes EC Talbi C Larrous F Dacheux L Bouchier C Bourhy H Genomic diversity and evolution of the lyssaviruses PLoS One 2008 303 4 e2057 18446239 10.1371/journal.pone.0002057 PMC2327259

[B5] Le MP Jacob Y Tordo N The complete Mokola virus genome sequence: structure of the RNA-dependent RNA polymerase J Gen Virol 1997 78 1571 1576 9225031 10.1099/0022-1317-78-7-1571

[B6] Nadin-Davis SA Abdel-Malik M Armstrong J Wandeler AI Lyssavirus P gene characterisation provides insights into the phylogeny of the genus and identifies structural similarities and diversity within the encoded phosphoprotein Virology 2002 298 2 286 305 10.1006/viro.2002.1492 12127791

[B7] Gerard FC Ribeiro EAJ Leyrat C Ivanov I Blondel D Longhi S Ruigrok RW Jamin M Modular organization of rabies virus phosphoprotein J Mol Biol 2009 388 5 978 996 10.1016/j.jmb.2009.03.061 19341745

[B8] Schnell MJ McGettigan JP Wirblich C Papaneri A The cell biology of rabies virus: using stealth to reach the brain Nat Rev Microbiol 2010 8 1 51 61 19946287 10.1038/nrmicro2260

[B9] Gupta AK Blondel D Choudhary S The phosphoprotein of rabies virus is phosphorylated by a unique celluar protein kinase and specific isomers of protein kinase C Virology 2000 74 1 91 98 10.1128/JVI.74.1.91-98.2000 PMC111517 10590095

[B10] Chenik M Schnell M Conzelmann KK Blondel D Mapping the interacting domains between the rabies virus polymerase and phosphoprotein J Virol 1998 72 1925 1930 9499045 10.1128/jvi.72.3.1925-1930.1998 PMC109484

[B11] Fu ZF Zheng Y Wunner WH Both the N and C- terminal domains of the nominal phosphoprotein of rabies virus are involved in binding to the nucleoprotein Virology 1994 200 2 590 597 10.1006/viro.1994.1222 8178445

[B12] Mavrakis M Méhouas S Réal E Iseni F Blondel D Tordo N Ruigrok RW Rabies virus chaperone: identification of the phosphoprotein peptide that keeps nucleoprotein soluble and free from non-specific RNA Virology 2006 349 2 422 429 10.1016/j.virol.2006.01.030 16494915

[B13] Green TJ Macpherson S Qiu S Lebowitz J Wertz GW Luo M Study of the assembly of vesicular stomatitis virus N protein: role of the P protein J Virol 2000 74 9515 9524 10.1128/JVI.74.20.9515-9524.2000 11000221 PMC112381

[B14] Jacob Y Real E Tordo N Functional interaction map of lyssavirus phosphoprotein: identification of the minimal transcription domains J Virol 2001 75 9613 9622 10.1128/JVI.75.20.9613-9622.2001 11559793 PMC114532

[B15] Schoehn G Iseni F Mavrakis M Blondel D Ruigrok RWH Structure of recombinant rabies virus nucleoprotein-RNA complex and identification of the phosphoprotein binding site J Virol 2001 75 490 498 10.1128/JVI.75.1.490-498.2001 11119617 PMC113941

[B16] Jacob Y Badrane H Ceccaldi PE Tordo N Cytoplasmic dynein LC8 interacts with lyssavirus phosphoprotein J Virol 2000 74 10217 10222 10.1128/JVI.74.21.10217-10222.2000 11024152 PMC102062

[B17] Raux H Flamand A Blondel D Interaction of the rabies virus P protein with the LC8 dynein light chain J Virol 2000 74 10212 10216 10.1128/JVI.74.21.10212-10216.2000 11024151 PMC102061

[B18] Lo KW Naisbitt S Fan JS Sheng M Zhang M The 8-kDa dynein light chain binds to its targets via a conserved (K/R)XTQT motif J Biol Chem 2001 276 14059 14066 11148209 10.1074/jbc.M010320200

[B19] Brzozka K Finke S Conzelmann KK Identification of the rabies virus alpha/beta interferon antagonist: phosphoprotein P interferes with phosphorylation of interferon regulatory factor 3 J Virol 2005 79 7673 7681 10.1128/JVI.79.12.7673-7681.2005 15919920 PMC1143667

[B20] Vidy A Chelbi-Alix MK Blondel D Rabies virus P protein interacts with STAT1 and inhibits interferon signal transduction pathways J Virol 2005 79 14411 14420 10.1128/JVI.79.22.14411-14420.2005 16254375 PMC1280226

[B21] Vidy A Bougrini J Chelbi-Alix MK Blondel D The nucleocytoplasmic rabies virus P protein counteracts interferon signaling by inhibiting both nuclear accumulation and DNA binding of STAT1 J Virol 2007 81 4255 4263 10.1128/JVI.01930-06 17287281 PMC1866157

[B22] Bernardi R Pandolfi PP Structure, dynamics and functions of promyelocytic leukaemia nuclear bodies Nature Rev Mol Cell Biol 2007 8 1006 1016 10.1038/nrm2277 17928811

[B23] Blondel D Kheddache S Lahaye X Dianoux L Chelbi-Alix MK Resistance to rabies virus infection conferred by the PMLIV isoform J Virol 2010 84 20 10719 10726 10.1128/JVI.01286-10 20702643 PMC2950589

[B24] Chenik M Chebli K Blondel D Translation initiation at alternate in-frame AUG codons in the rabies virus phosphoprotein mRNA is mediated by a ribosomal leaky scanning mechanism J Virol 1995 69 707 712 7815533 10.1128/jvi.69.2.707-712.1995 PMC188632

[B25] Bourhy H Reynes JM Dunham EJ Dacheux L Larrous F Huong VT Xu G Yan J Miranda ME Holmes EC The origin and phylogeography of dog rabies virus J Gen Virol 2008 89 2673 2681 10.1099/vir.0.2008/003913-0 18931062 PMC3326349

[B26] Tao XY Tang Q Li H Mo ZJ Zhang H Wang DM Zhang Q Song M Velasco-Villa A Wu XF Rupprecht CE Liang GD Molecular epidemiology of rabies in southern People’s Republic of China Emerg Infect Dis 2009 15 1992 1998 10.3201/eid1508.081551 PMC2815963 19751579

[B27] Ming PG Yan JX Simon R Meng SL Xu GL Tang Q Wu J Luo J Yang XM A history estimate and evolutionary analysis of rabies virus variants in China J Gen Virol 2010 91 759 764 10.1099/vir.0.016436-0 19889927

[B28] Meng SL Yan JX Xu GL Nadin-Davis SA Ming PG Liu SY Ming HT Zhu FC Zhou DJ A molecular epidemiological study targeting the glycoprotein gene of rabies virus isolates from China Virus Res 2007 124 125 138 10.1016/j.virusres.2006.10.011 17129631

[B29] Conzelmann K Cox JH Schneider LG Thiel H Molecular cloning and complete nucleotide sequence of the attenuated rabies virus SAD B19 Virology 1990 175 485 499 10.1016/0042-6822(90)90433-R 2139267

[B30] Larson JK Wunner WH Nucleotide and deduced amino acid sequences of the nominal nonstructural phosphoprotein of the ERA, PM and CVS-11 strains of rabies virus Nucleic Acids Res 1990 18 23 7172 10.1093/nar/18.23.7172 2148206 PMC332818

[B31] Nadin-Davis S Huang W Wandeler AI Polymorphism of rabies viruses within the phosphoprotein and matrix protein genes Arch Virol 1997 142 1 14 10.1007/s007050050055 9191862

[B32] Gould A Hyatt AD Lunt R Kattenbelt JA Hengstberger S Blacksell SD Characterisation of a novel lyssavirus isolated from pteropid bats in Australia Virus Res 1998 54 165 187 10.1016/S0168-1702(98)00025-2 9696125

[B33] Bourhy H Bachir K Tordo N Molecular diversity of the Lyssavirus genus Virology 1993 194 70 81 10.1006/viro.1993.1236 8386891

[B34] Banerjee AK Barik S Gene expression of vesicular stomatitis virus genome RNA Virology 1992 188 417 428 10.1016/0042-6822(92)90495-B 1316668

[B35] Gong WJ Jiang Y Zhang YF Zeng Z Shao MF Fan JH Sun YW Xiong ZL Yu XL Tu CC Temporal and spatial dynamics of rabies viruses in China and Southeast Asia Virus Res 2010 150 111 118 10.1016/j.virusres.2010.02.019 20214936

[B36] Bourhy H Kissi B Audry L Smreczak M Sadkowska-Todys M Kulonen K Tordo N Zmudzinski JF Holmes EC Ecology and evolution of rabies virus in Europe J Gen Virol 1999 80 2545 2557 10573146 10.1099/0022-1317-80-10-2545

[B37] Davis PL Bourhy H Holmes EC The evolutionary history and dynamics of bat rabies virus Infect Genet Evol 2006 6 464 473 10.1016/j.meegid.2006.02.007 16621724

[B38] Talbi C Holmes EC de Benedictis P Faye O Nakoune E Gamatie D Diarra A Elmamy BO Sow A Adjogoua EV Sangare O Dundon WG Capua I Sall AA Bourhy H Evolutionary history and dynamics of dog rabies virus in western and central Africa J Gen Virol 2009 90 783 791 10.1099/vir.0.007765-0 19264663

[B39] Badrane H Tordo N Host switching in lyssavirus history from the Chiroptera to the Carnivora orders J Virol 2001 75 8096 8104 10.1128/JVI.75.17.8096-8104.2001 11483755 PMC115054

[B40] Zhang YZ Xiong CL Lin XD Zhou DJ Jiang RJ Xiao QY Xie XY Yu XX Tan YJ Li M Ai Q Zhang LJ Zou Y Huang C Fu ZF Genetic diversity of Chinese rabies viruses: Evidence for the presence of two distinct clades in China Infect Genet Evol 2009 9 87 96 10.1016/j.meegid.2008.10.014 19041424

[B41] Yamada K Ito N Takayama-Ito M Sugiyama M Minamoto N Multigenic relation to the attenuation of rabies virus Microbiol Immunol 2006 50 25 32 16428870 10.1111/j.1348-0421.2006.tb03767.x

[B42] Shimizu K Ito N Mita T Yamada K Hosokawa-Muto J Sugiyama M Minamoto N Involvement of nucleoprotein, phosphoprotein, and matrix protein genes of rabies virus in virulence for adult mice Virus Res 2007 123 154 160 10.1016/j.virusres.2006.08.011 17010466

[B43] Thompson JD Gibson TJ Plewniak F Jeanmougin F Higgins DG The ClustalX windows interface: flexible strategies for multiple sequence alignment aided by quality analysis tools Nucleic Acids Res 1997 25 4876 4882 10.1093/nar/25.24.4876 9396791 PMC147148

[B44] Hall TA BioEdit: a user-friendly biological sequence alignment editor and analysis program for Windows 95/98/NT Nucleic Acids Symp 1999 41 95 98

[B45] Kumar S Tamura K Nei M MEGA3: integrated software for molecular evolutionary genetics analysis and sequence alignment Brief Bioinform 2004 5 50 163 10.1093/bib/5.2.150 15260895

[B46] Kimura M A simple method for estimating evolutionary rates of base substitutions through comparative studies of nucleotide sequences J Mol Evol 1980 15 111 120 7463489 10.1007/BF01731581

[B47] Tajima F Nei M Estimation of evolutionary distance between nucleotide sequences Mol Biol Evol 1984 1 269 285 6599968 10.1093/oxfordjournals.molbev.a040317

[B48] Drummond AJ Rambaut A BEAST: Bayesian evolutionary analysis by sampling trees BMC Evol Biol 2007 7 214 10.1186/1471-2148-7-214 17996036 PMC2247476

[B49] Posada D Crandall KA MODELTEST: testing the model of DNA substitution Bioinformatics 1998 14 9 817 818 10.1093/bioinformatics/14.9.817 9918953

[B50] Drummond AJ Ho SYW Phillips MJ Rambaut A Relaxed phylogenetics and dating with confidence PLoS Biol 2006 4 e88 10.1371/journal.pbio.0040088 16683862 PMC1395354

